# The Aging Eye

**DOI:** 10.1155/2015/832326

**Published:** 2015-03-24

**Authors:** Suddhasil Mookherjee, Ashima Bhattacharjee, Mainak Sengupta

**Affiliations:** ^1^NEI, NIH, Bethesda, MD, USA; ^2^Johns Hopkins University School of Medicine, Baltimore, MD, USA; ^3^Department of Genetics, University of Calcutta, Kolkata, India

With the advancement of medical science, average life expectancy of individuals is ever increasing. Percentage of elderly population has increased from 6.2% in 1990 to 8.2% in 2015 worldwide (http://www.unescap.org/resources/1-population). However, with increasing life expectancy, the prevalence of age related diseases will also increase, which will affect the quality of life in elderly population worldwide.

Eye is our window to the outer world and deterioration of this vital organ with age will definitely affect the lifestyle of the elderly people. According to WHO, approximately 65% of the population aged over 50 years has some forms of visual impairment (http://www.who.int/mediacentre/factsheets/fs282/en/). Therefore, understating and managing the age related changes in eye calls for significant effort and attention from the scientific world. This special issue focuses on the age related changes and diseases of eye as well as their management in elderly population. Eight meticulously chosen papers in this special issue focus on different aspects of vision problem and their management in elderly population.

Cataract is one of the major eye pathologies of elderly people. X. Yuan et al. reported the prevalence of astigmatism in eyes requiring cataract surgery. They found that prevalence of astigmatism also increases with age and discussed its implication in visual rehabilitation. L. Zuo et al. discussed the effect of unilateral or bilateral cataract surgery on visual acuity and quality of life in elderly patient.

Retinal pigment epithelium (RPE) is involved in various age related pathological changes in the eye including AMD. A. V. Kuznetsova et al. discussed the prospects of using RPE cell line in modelling various pathological changes in human disease and their potential use in retinal repair after ocular pathologies. They also discussed various signaling pathways in RPE which can be used as potential targets for different eye diseases.

L. Ottobelli et al. discussed the changes in ocular surface with increasing age and determined the frequency of ocular surface disease (OSD) with age. They concluded that dry eye disease (DED) is the most frequently occurring OSD and its frequency increases with age. A. Sharma and H. B. Hindman reviewed various aspect of DED with the increasing age.

Glaucoma is another major cause of vision loss especially in elderly population. Early detection and medical intervention are necessary to control vision loss due to glaucoma. Measurement of anterior chamber angle is an important parameter for diagnosis and management of glaucoma. M. Rigi et al., in this issue, have described a new parameter called TICV (trabecular-iris circumference volume) to detect the health of anterior chamber by optical coherence tomography. N. L. Pratt et al. discussed the relationship between bradycardia and Timolol, a widely used glaucoma medication, and concluded that patients' medical history should be reviewed before prescribing the medication. The study emphasizes the importance of considering full clinical information of patients for deciding treatment regimen taking into account other systemic ailments that the patient is already exposed to or might be predisposed to.

Finally, M. Oles and P. Oles discussed the issue of quality of lifestyle in people with low vision especially due to cataract and glaucoma. Their study highlights the importance of social support and task oriented coping to improve quality of life for individuals with low vision.

Age related eye diseases, being one of the most prevalent causes of blindness worldwide, demand attention as a global health problem. Research efforts must be concentrated on early detection and cost-effective methods of intervention accessible by people belonging to all economic strata globally. This issue is a small step towards such efforts for prevention of blindness and improving quality of human lives with progression of age.

*Suddhasil Mookherjee* *Suddhasil Mookherjee* *Ashima Bhattacharjee* *Ashima Bhattacharjee* *Mainak Sengupta* *Mainak Sengupta*

